# Non-cultured dermal-derived mesenchymal cells attenuate sepsis induced by cecal ligation and puncture in mice

**DOI:** 10.1038/srep16973

**Published:** 2015-11-20

**Authors:** Yu Wang, Li Tan, Jie Jin, Huiqin Sun, Zelin Chen, Xu Tan, Yongping Su, Chunmeng Shi

**Affiliations:** 1 Institute of Combined Injury, State Key Laboratory of Trauma, Burns and Combined Injury, Chongqing Engineering Research Center for Nanomedicine, College of Preventive Medicine, Third Military Medical University, 30 Gaotanyan Road, Chongqing 400038, China; 2 Department of Hematology, Daping Hospital, Third Military Medical University, 10# Daping Changjiang Road, Chongqing, 400042, China

## Abstract

Sepsis remains a threat to critically ill patients and carries a high morbidity and mortality. Cell–based therapies have risen in prominence in recent years. Dermal-derived mesenchymal cells (DMCs) are attractive as one of the abundant sources from which to isolate mesenchymal cells for therapeutic applications and can be easily accessed with minimal harm to the donor. In this study, we described for the first time the use of non-cultured DMCs for treating sepsis in a cecal ligation and puncture (CLP) mouse model and investigated their immunomodulatory effects. We found that non-cultured DMCs administration provides a beneficial effect to improve survival in CLP-induced sepsis. This effect is partly mediated by the ability of DMCs to home to sites of injury, to reduce the inflammatory response, to inhibit apoptosis, and to stimulate macrophage migration and phagocytosis. Our further findings suggest that DMCs treatment modulates the beneficial cytoprotective effects exhibited during sepsis, at least in part, by altering miRNA expression. These discoveries provide important evidence that non-cultured DMCs therapy has a specific anti-inflammatory effect on sepsis, and provide the basis for the development of a new therapeutic strategy for managing clinical sepsis.

The sepsis syndrome is defined by widespread inflammation, host immune dysfunction, dysregulation of the coagulation cascade, and endothelial dysfunction in response to invading pathogens. It represents a continuum of diseases, led by systemic inflammatory response syndrome (SIRS). This may then progress to septic shock, and significant multiple organ dysfunction, potentially resulting in death^1^. It is reported that the prevalence of sepsis is estimated at approximately 0.3% of the total population, and about 18 million cases occur each year worldwide. The mortality from sepsis amounts to as high as about 28% ~ 50%, with an average of 40%^2^. In the United States, the average cost of treatment per patient is about $ 22,000, and the total cost reaches nearly $ 20 billion per year^3^,^4^. Sepsis has constituted a serious threat to human health and a huge burden to economic development.

Although numerous studies on the pathogenesis of sepsis and its clinical treatment have been conducted, many problems have not been fundamentally resolved, and mortality from sepsis remains high. Some once common treatment, such as anti-tumor necrosis factor (TNF)-α antibodies^5^,^6^, interleukin (IL)-1 receptor antagonists^7^, and platelet-activating factor (PAF) antagonists^8^,^9^, etc., fails to improve the cure and survival rates of sepsis. Single-target molecular therapies have not addressed the multiple disease pathways triggered by septic injury. But cells are more versatile than molecules: They make products and respond to their environments^10^. Mesenchymal stromal/stem cells (MSCs) were administrated to patients with severe acute graft-versus-host diseases and positive effects were obtained^11^. Xu J *et al.*^12^ found that bone marrow-derived MSCs could decrease both the systemic and local inflammatory responses induced by endotoxin. Gupta N *et al.*^13^ demonstrated that intrapulmonary delivery of bone marrow-derived MSCs improved survival and attenuated endotoxin-induced acute lung injury in mice. Compared to MSCs, different site-specific mesenchymal cells also show immunomodulatory properties for a cell therapy candidate. Recently, Fletcher *et al.* demonstrated that murine lymph node fibroblastic reticular cells effectively reduced death from sepsis, inhibited key proinflammatory cytokines in blood and peritoneum, and reduced bacterial load. Skin is the largest organ in the body and has long been researched for its use as a potential source of regenerative cells. With this regard, dermal-derived mesenchymal cells (DMCs) are attractive as one of the abundant sources from which to isolate mesenchymal cells for therapeutic applications and can be easily accessed with minimal harm to the donor. Additionally, recent studies have demonstrated that *in vitro* culture may alter the functional activities of cells and it is believed that cells isolated from the natural microenvironment can better mimic their properties *in vivo* than the commonly used 2D cultures^14^. Moreover, for some patients, such as severe burns and sepsis, they require an off the shelf product that is immediately available without *in vitro* culture. We previously proved that non-cultured mouse DMCs decreased the incidence and severity of acute GVHD during MHC-mismatched stem cell transplantation in mice^15^. In this study, we aim to test the therapeutic effect and mechanism of non-cultured DMCs in CLP-indcuced sepsis in mice and investigate their potential mechanism of immunomodulatory effects.

## Materials and Methods

### Mice

C57BL/6J male mice (8–12 wk old) were maintained in the animal facility at Third Military Medical University (TMMU). All experimental protocols were approved by the Institutional Animal Care and Use Committee of TMMU, and were conducted in accordance with the “Animal Care and Use Committee Guidelines” of the university.

### Cell preparation

DMCs preparation: The procedures of DMCs preparation were performed as previously described^15^. In brief, full-thickness skin samples were obtained from the dorsum of neonatal C57 mice, digested at 4 °C overnight with 0.25% trypsin (Hyclone). Next, the dermis layer was dissociated by flushing with D-Hank’s solution, and the suspension was filtered through a nylon mesh to remove cellular debris and centrifuged. Finally, 1 × 10^7^ DMCs could be isolated from the dermis of one neonatal C57 mouse, and then they were infused intravenously without cultivation and proliferation *in vitro*.

Peritoneal macrophages preparation: Peritoneal macrophages were cultured in 6-well plates in RPMI-1640 (Gibco) containing 1% FCS (Gibco). The next day, plates were washed with cold phosphate-buffered saline (PBS) for 3 times, nonadherent cells were discarded, and adherent cells were cultured in DMEM-H (Gibco) with 10% FCS for further use.

### Survival study and histology assessment of mice with CLP-induced sepsis treated with non-cultured DMCs

The CLP model was used to induce intra-abdominal peritonitis^16^. The mice were anesthetized with sodium pentobarbital, and an abdominal incision was performed. The cecum was identified, ligated on medium cecal, and punctured twice with a #21-gauge needle. A small amount of fecal material was gently squeezed and then the cecum was returned to the central abdominal cavity. The abdomen was thus closed in two layers. After surgery, we administered pre-warmed normal saline subcutaneously by 1 ml per 30 g body weight. Sham-operated mice were treated in the same manner, except that the cecum was not ligated or punctured. Saline or DMCs (at 2 × 10^6^ cells, total volume of 100 μl) were slowly infused via the tail vein 4 hours after sham operation or CLP. Additional experiments were done with a mouse fibroblast cell line (3T3, at 2 × 10^6^ cells, total volume of 100 μl) and also saline (100 μl) injected through the tail vein as two separate controls. The mice were randomized to receive saline, 3T3 fibroblasts or DMCs. Their body temperature was maintained at 37 °C using a heating pad after operation. We placed mice back in cages in a temperature-controlled room (22 °C) with 12-h light and dark cycles immediately after surgery. We monitored survival rate, activity, and defecation of mice every 6 hours for the first 24 hours, and thereafter every 12 hours for the following 8 days. The ileocecal region from both the DMCs group and the control group were excised 7 days after CLP-induced sepsis. Then, sections were fixed with 4% paraformaldehyde. Hematoxylin and eosin staining was performed for histopathology. Lungs, liver, spleen and ileocecal region of each group were taken 48 h after CLP surgery or sham operations for apoptosis analysis. Apoptosis was assessed by Terminal deoxynucleotidyl transferase dUTP Nick End Labeling (TUNEL) staining with *in situ* cell death detection kit (Roche, Penzberg, Germany).

### DMCs labeling and whole-body animal NIR fluorescence imaging

To track cell homing patterns *in vivo*, DMCs were labeled with IR-780 iodide before injection, and tracked for 8 days thereafter. Briefly, for each animal 2 × 10^6^ cells were labeled with 20 μM IR-780 iodide for 15 min at 37 °C according to previous protocols^17^ under sterile conditions. IR-780 iodide-labeled DMCs were injected via the tail vein 4 hours after operation. Then NIR imaging of small animals were conducted using Kodak In-Vivo FX Professional Imaging System. Mice were anesthetized with pentobarbital and maintained in an anesthetized state during the imaging procedure. The mice administered with IR-780 iodide-labeled DMCs were shaved and then imaged at time points of 1 d, 4 d, and 8 d after injection, respectively.

After imaging, the mice were sacrificed, and organs were harvested from mice and fixed by 4% paraformaldehyde for 24 hours. Next, the organs were embedded in optimal cutting temperature compound (OCT) (Zsbio, Beijing, China), snap-frozen, and cryo-sectioned at 8 μm. Sections were stained with DAPI and then examined by laser scanning confocal microscopy (Carl Zesis).

### Co-culture of Lipopolysaccharide-stimulated Macrophage and DMCs

To determine whether DMCs cells were able to regulate production of inflammatory cytokines by stimulated macrophage, by a cell-cell contact-dependent or -independent mechanism, peritoneal macrophages or macrophages Raw264.7 were co-cultured with DMCs in either a standard single well or a Transwell (pore size of 0.4 μm; Costar; Cambridge, Massachusetts, USA) and then stimulated with LPS^18^. First, we plated 0.6 ml of DMCs into the lower chamber of 24-well transwell at a concentration of 2 × 10^5^/ml. After 2 h, we added 0.2 ml of macrophages to the upper chamber of 24-well transwell at a concentration of 2 × 10^5^/ml. As a control, we plated macrophages without DMCs. After an overnight incubation, we added LPS to the co-cultures to reach a final concentration of 1 μg/ml. We collected the supernatants 1 and 2 d after the stimulation respectively, spun them down to remove possible cell contamination (3,000 rpm for 10 min) and stored the resulting supernatants at −80 °C for further use.

### Cytokine measurement

Cell supernatants were collected and centrifuged at 3,000 rpm for 10 min to remove possible cell contamination and stored at −80 °C until further examination. Retro-orbital blood were collected under anesthesia at 24 h after CLP. Blood samples were centrifuged at 3,000 rpm for 20 min, and the supernatants were collected and stored at −80 °C until further test. Cytokine concentrations were measured by enzyme-linked immunosorbent assay (ELISA). IL-1β, IL-6, and IL-10 levels in the supernatants of LPS-stimulated cells and IL-1β, IL-6, IL-4, IL-5, IL-10 and IFN-γ levels in the mice circulating (serum) were determined using specific ELISA kits (CUSABIO). we did a pre-test to determine the optimal sample diluted concentration, then we added 100ul diluted sample per well to measure cytokines expression according to the manufacturer’s instructions. Each sample was run in duplicate.

### Apoptosis analysis

To determine whether DMCs cells were able to decrease apoptosis of stimulated peritoneal macrophages or Raw264.7 cells, macrophages were co-cultured with DMCs in a Transwell (pore size of 0.4 μm; Costar; Cambridge, Massachusetts, USA) and then stimulated with LPS. For macrophages apoptosis quantification, flow cytometry was performed using a commercially available annexin V-fluorescein isothiocyanate (FITC) apoptosis detection kit according to the manufacturer’s guidelines (BD Pharmingen, San Diego, Calif., USA). In brief, macrophages were cultured in the transwell lower chamber, and DMCs were cultured in the transwell upper chamber. After an overnight incubation, we added LPS to the co-cultures to reach a final concentration of 1 μg/ml. After LPS stimulation for 24 h, the upper chamber was removed, and macrophages in the lower chamber was isolated with enzymatic digestion and centrifuged. The cell pellets were resuspended in the kit binding buffer and then centrifuged again. The pellets were then resuspended in kit buffer (100 μl/pellet) containing annexin V solution (5 μl/pellet) and propidium iodide (2.5 μg/pellet). After incubation in the dark for 10 min, the percentage of annexin V-positive cells was measured with a FACSCanto II flow cytometer (Becton Dickinson BD).

### Migration assay

To determine whether DMCs cells were able to regulate the migration activity of macrophages in the presence of LPS stimulation, peritoneal macrophages or macrophages Raw264.7 were co-cultured with DMCs in a 24-well Transwell (pore size of 8 μm; Costar; Cambridge, Massachusetts, USA) or cultured alone, and then stimulated with LPS or not. Briefly, we plated 0.6 ml of DMCs into the lower chamber of 24-well transwell at a concentration of 2 × 10^5^/ml. After 2 h, we added 0.2 ml of macrophages to the upper chamber of 24-well transwell at a concentration of 2 × 10^5^/ml. As a control, we plated macrophages without DMCs. After 4 h co-culture incubation, in one group, we added LPS to the co-cultures to reach a final concentration of 1 μg/ml. In another group, we did not stimulate the cell by LPS. Cells were allowed to migrate for 6 h at 37 °C in 5% CO_2_. Migrated cells, attached to the lower face of the porous membrane, were fixed with paraformaldehyde for 20 min, and stained with Hematein for 3 min. Unmigrated cells on the upper membrane surface were removed with a cotton swab. The migration was quantified by analyzing at least 5 random fields per filter for each independent experiment.

### Phagocytosis Assay

Phagocytosis analysis *in vitro*: To determine whether DMCs cells were able to stimulate phagocytosis of stimulated peritoneal macrophages or macrophages Raw264.7, macrophages were co-cultured with DMCs in a 6-well plates transwell (pore size of 0.4 μm; Costar; Cambridge, Massachusetts, USA). As a control, we plated macrophages without DMCs. After 4 h co-culture incubation, we added LPS to the co-cultures to reach a final concentration of 1 μg/ml. and then peritoneal macrophages were incubated with fluorescent latex beads (Sigma, L-3030, 5 μL beads in 2 mL of DMEM-H) for 4 h, and macrophages Raw264.7 were incubated with fluorescent latex beads for 12 h. For microscopic analysis, macrophages were analyzed by fluorescence microscope (Carl Zesis). For flow cytometry analysis, macrophages were treated as described above and detached by cell scraper after washing in PBS. Peritoneal macrophages were identified using a PE anti-mouse F4/80 antibody (BioLegend) and the fluorescence was measured by FACS Calibur flow cytometer (Becton-Dickenson, San Jose, CA).

Phagocytosis analysis *in vivo*: The CLP mice were made and treated with saline or DMCs as described above. One day after CLP, fluorescent latex beads (Sigma, L-3030, 10 μL beads in total volume of 100 μl) were injected into the peritoneal cavity of CLP mice. Mice were sacrificed 2 hours after injection. Peritoneal cells were stained as described above and analyzed by FACS.

### Murine peritoneal macrophages MiRNA isolation and MiRNA microarray

Murine peritoneal macrophages were prepared 24 h after CLP surgery or sham operations. Briefly, One day after CLP surgery, macrophages were harvested from the peritoneal cavity by lavaging with RPMI culture media. The macrophages were centrifuged, total RNA of murine peritoneal macrophages was harvested using TRIzol (Invitrogen) and miRNeasy mini kit (QIAGEN) according to manufacturer’s instructions. After having passed RNA quantity measurement using the NanoDrop 1000, the samples were labeled using the miRCURY™ Hy3™/Hy5™ Power labeling kit and hybridized on the miRCURY™ LNA Array (v.18.0). Following the washing steps the slides were scanned using the Axon GenePix 4000B microarray scanner. Scanned images were then imported into GenePix Pro 6.0 software (Axon) for grid alignment and data extraction. Replicated miRNAs were averaged and miRNAs that intensities > = 30 in all samples were chosen for calculating normalization factor. Expressed data were normalized using the Median normalization. After normalization, differentially expressed miRNAs were identified through Fold Change filtering. Finally, hierarchical clustering was performed to show distinguishable miRNA expression profiling among samples.

### Bioinformatics analysis using gene ontology and the Kyoto Encyclopedia of Genes and Genomes

We generated predicted mRNA targets for select miRNAs using TargetScan, MicroCosm Targets, and miRanda. The intersection of these datasets was taken as reliable. Gene ontology (GO) category and Kyoto Encyclopedia of Genes and Genomes (KEGG) pathway analyses of target genes of differentially expressed miRNAs were performed using the web-based tool, Database for Annotation, Visualization, and Integrated Discovery (DAVID, http://david.abcc.ncifcrf.gov/)^19^,^20^. We used two-sided Fisher’s exact test and the chi-square test to select significant pathways, and defined the threshold of significance as P < 0.01 and the false discovery rate as <0.01.

### Statistical analysis

Data were expressed as mean ± standard deviation. Survival data were compared using the Kaplan-Meier test (Prism 4.0; Graphpad Software).An independent-samples t test was used to determine the significant differences between two groups. P < 0.05 was considered statistically significant. Bioinformatics analysis were performed as described above.

## Results

### Non-cultured DMCs treatment improved the survival after CLP in mice

**Figure 1:**
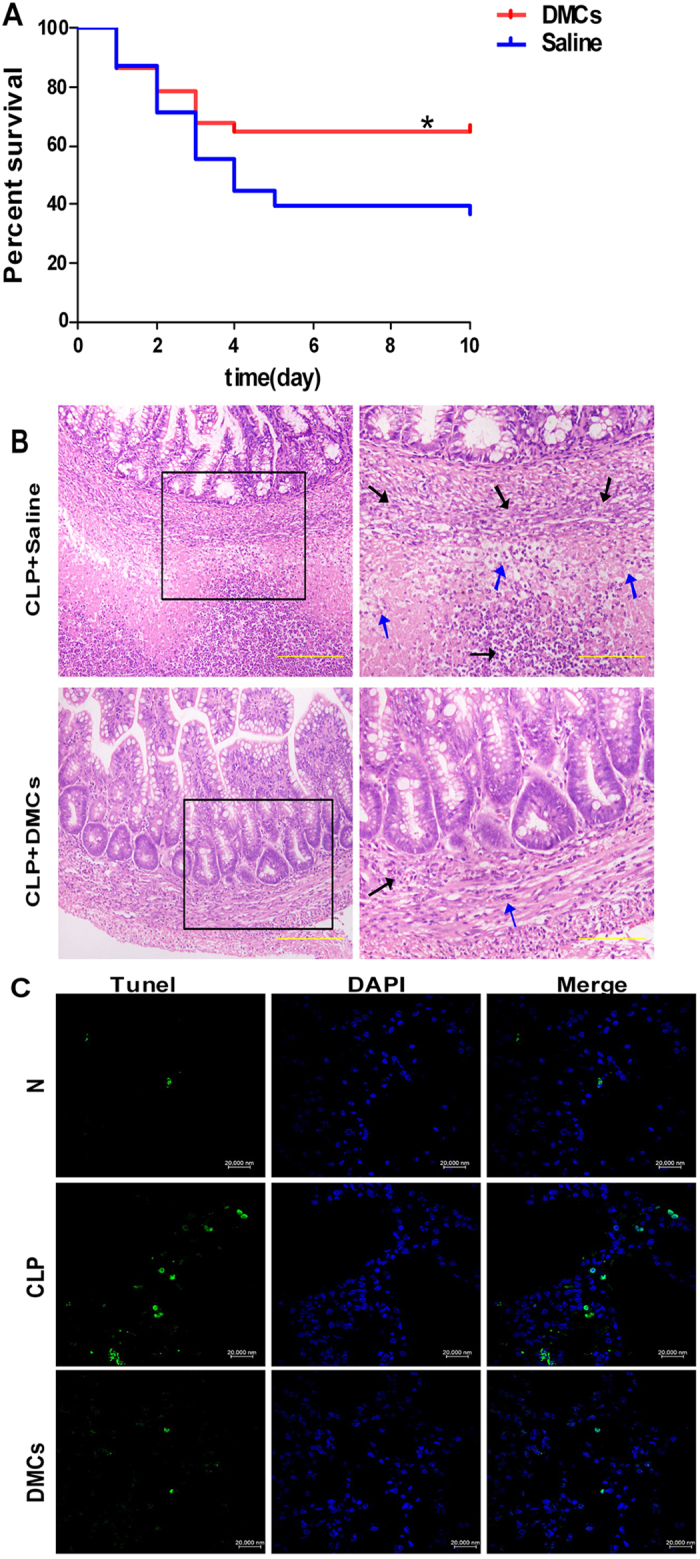
Non-cultured DMCs treatment improved the survival and decreased intestinal damage after CLP in mice (A) Non-cultured DMCs treatment significantly reduced mortality in septic mice. Survival rate following CLP -induced sepsis in mice treated with DMCs (n = 37) or saline (n = 38). Kaplan-Meier curves represent survival rate in the 2 groups. Survival rate was significantly higher in the DMCs group than the saline groups: *P < 0.05, DMCs vs. saline. (**B**) Hematoxylin and eosin were used to stain ileocecal region tissues from representative animals 7 days after CLP. Transplantation of DMCs reduced inflammatory cell infiltration (indicated by black arrowhead) and exudation (indicated by blue arrowhead) after CLP. The ileocecal region was fixed with 4% paraformaldehyde, embedded in paraffin, and cut into 5-μm-thick sections before stained. (**C**) Apoptosis was assessed by TUNEL staining 2 days after CLP. CLP -induced sepsis also increased the percentage of apoptotic cells in the lung, and treatment with DMCs reduced apoptotic cells to the levels observed in sham-operated animals.

We have established DMCs with multilineage differentiation capacity and confirmed that about 72.6% cells express surface marker CD105. Here, we showed that the survival rate was significantly increased in the mice treated with non-cultured DMC compared with those treated with saline. The survival rate at 10 d was 64.9% in the CLP/DMCs group and 36.8% in the CLP/saline group (Fig. 1A). In contrast, intravenous injection of 3T3 fibroblasts did not alter survival compared with CLP/saline group (30% vs. 36.8% at 10 d). Histologic examination revealed severe injuries in the intestinal mucosa, including apparent edema, necrosis of intestinal villi, degeneration of epithelial cells and infiltration of inflammatory cells in the CLP/saline group, whereas in the CLP/DMCs group appeared mild edema and reduced inflammatory cell infiltration in the ileocecal region after CLP (Fig. 1B). Sepsis also increased the percentage of apoptotic cells in the lung, and treatment with DMCs reduced apoptotic cells to the levels observed in sham-operated animals (Fig. 1C).

### Effect of DMCs on blood cytokine concentrations

**Figure 2:**
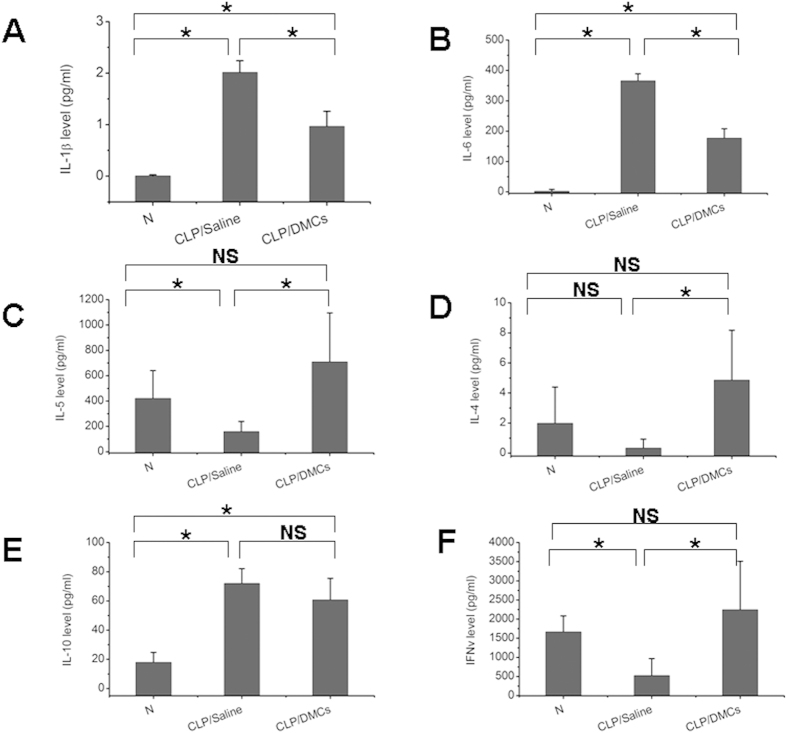
Non-cultured DMCs can modulate serum cytokine secretion after CLP operation. Sepsis was induced in male C57Bl/6J mice by cecal ligation and puncture (CLP). Saline or a suspension of non-cultured DMCs (2 × 10^6^ cells in 100 μl of saline) was slowly infused via the tail vein 4 hours after CLP operation. Serum concentrations of IL-1β, IL-6, IL-5, IL-4, IL-10 and IFN-γ were measured after CLP or CLP and DMCs treatment. Serum concentrations of IL-1β (**A**), IL-6 (**B**), IL-5 (**C**), IL-4 (**D**), IL-10 (**E**) and IFN-γ (**F**) at 24h after CLP. n = 5–6 at each group. Error bars represent means ± SD. * indicates Independent-Samples t-test p-values < 0.05. NS means no significant difference.

We hypothesized that DMCs might alter the immune response to infection. Therefore, we investigated blood IL-1β, IL-6, IL-4, IL-5, IL-10 and IFN-γ, which play important roles in sepsis. At 24 hours after CLP was induced, IL-1β and IL-6 levels were attenuated from their pathogenic values in DMCs treated mice relative to saline treated control group (Fig. 2A,B). And blood IL-4, IL-5, and IFN-γ were increased in DMCs treated mice, comparing to saline treated control septic mice. Moreover, Serum IL-4, IL-5, and IFN-γ levels were close to wild-type levels (Fig. 2C,D,F) in DMCs treated relative to saline-treated control mice. No significant differences were observed in serum levels of the anti-inflammatory cytokine IL-10 between DMCs treated mice and saline treated control mice(Fig. 2E).

### Tracing and distribution of DMCs *in vivo*

**Figure 3:**
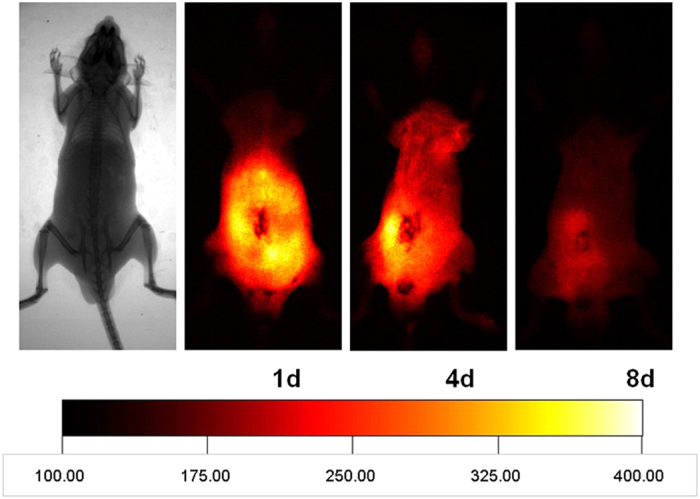
Migration of non-cultured DMCs administered intravenously in experimental animals. Ir780-labeled DMCs were administered to animals and traced for 8 days by whole-body animal NIR imaging. Ir780-labeled DMCs migrated towards the whole abdomen on day 1, and then concentrated on the inflamed ileocecal region in the left lower quadrant on day 4. After 8 days, the labeled cells were barely detectable. Images represent 3 independent experiments. All the animals are lying on their backs.

**Figure 4:**
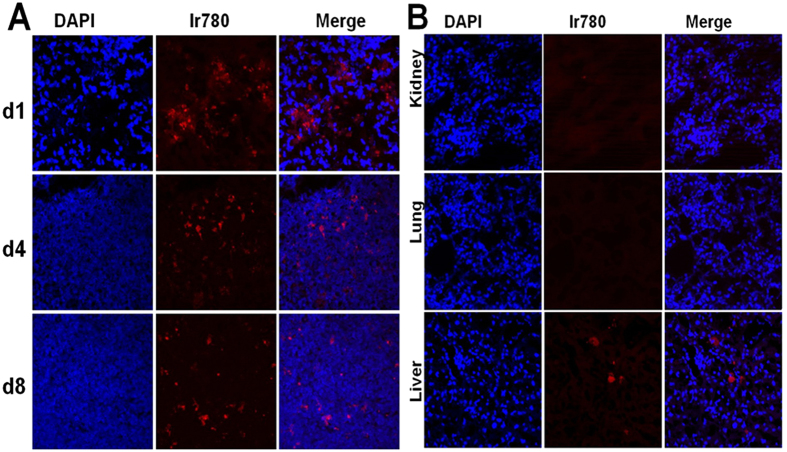
Distribution and engraftment of DMCs administered intravenously in the ileocecal region. Ir780-labeled DMCs (red) injected intravenously were tracked during the 8 days. After surgical removal of the ileocecal region, samples were cut on a cryostat, counterstained with DAPI (blue), and analyzed under a confocal microscope. Labeled cells migrated towards the inflamed ileocecal region (**A**), but rarely to the non-inflamed bowel and other organs (kidney, lung, and liver, (**B**) Ir780-labeled DMCs were observed predominantly in the abscess formed around the CLP site. Images represent 3 independent experiments.

Ir780-labeled DMCs were administered to animals via their tail vein and traced for 8 days by Kodak In-Vivo FX Professional Imaging System. IR780-labeled DMCs migrated towards the whole abdomen on day 1, and then concentrated on the inflamed ileocecal region in the left lower quadrant on day 4. After 8 days, labeled cells were barely detectable (Fig. 3). In contrast, labeled cells were barely detectable in sham control mice on day 4 (data not shown). To further evaluate the tissue distribution of intravenously injected DMCs which migrated towards the inflamed ileocecal region, we observed frozen organ sections by laser scanning confocal microscopy. The red Ir780-labeled DMCs were mainly observed in the subserosa of ligated cecum, predominantly in the abscess around the ligated site. DMCs were consistently distributed around the inflamed ileocecal region for more than 8 days (Fig. 4A). However, in the non-inflamed tissue such as the proximal colon, no labeled DMCs were observed (data not shown). We found labeled DMCs in neither lung nor kidney, and rarely labeled DMCs were found in the liver (Fig. 4B). Histological analysis of the organ tissue further revealed that most IR-780 iodide labeled DMCs were concentrated in the ligated ileocecal region, and the labeled DMCs were still detectable at day 8.

### Effect of DMCs on the secretion of macrophages supernatant cytokines

**Figure 5:**
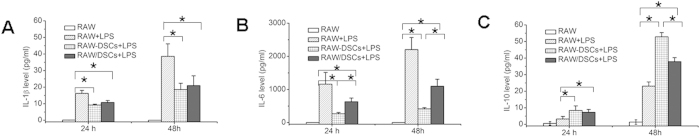
Effect of DMCs on the secretion of Raw264.7 cell supernatant cytokines. (**A**) The co-culture of DMCs with Raw264.7 decreased IL-1β secretion at both 24 and 48 h after LPS stimulation, whereas there was no significant difference in IL-1β secretion between contact or non-contact co-culture manner when compared to Raw264.7 alone. (**B**) The co-culture of DMCs with Raw264.7 decreased IL-6 secretion at both 24 and 48 h after LPS stimulation, and there were significant differences in IL-6 secretion between contact and non-contact co-culture manners when compared to Raw264.7 alone. (**C**) The co-culture of DMCs with Raw264.7 increased IL-10 secretion at both 24 and 48 h after LPS stimulation, and there were significant differences in IL-10 secretion between contact and non-contact co-culture manners when compared to Raw264.7 alone at 48 h, whereas no significant difference was observed at 24 h. RAW: Raw264.7 alone without LPS stimulation; RAW + LPS: Raw264.7 alone with LPS stimulation; RAW-DMCs + LPS: mixture contact co-culture of DMCs with Raw264.7 with LPS stimulation; RAW/DMCs + LPS: non-contact co-culture of DMCs with Raw264.7 with LPS stimulation. n = 5 at each group. * indicates Independent-Samples t-test p-values < 0.05.

The co-culture of DMCs with Raw264.7 decreased IL-1β secretion at both 24 and 48 h after LPS stimulation, whereas there was no significant difference in IL-1β secretion between contact or non-contact co-culture manner when compared to Raw264.7 alone (Fig. 5A). The co-culture of DMCs with Raw264.7 decreased IL-6 secretion at both 24 and 48 h after LPS stimulation, and there were significant differences in IL-6 secretion between contact or non-contact co-culture manner when compared to Raw264.7 alone (Fig. 5B). The co-culture of DMCs with Raw264.7 increased IL-10 secretion at both 24 and 48 h after LPS stimulation, and there were significant differences in IL-10 secretion between contact and non-contact co-culture manner when compared to Raw264.7 alone at 48 h, whereas no significant difference was observed at 24 h (Fig. 5C). Similar to the Raw264.7 supernatant cytokines assessments, in the peritoneal macrophages, we observed IL-6 were significantly decreased in co-culture group comparing to peritoneal macrophages alone after LPS stimulation.

### Co-culture with DMCs led to inhibition of macrophages apoptosis induced by LPS

**Figure 6:**
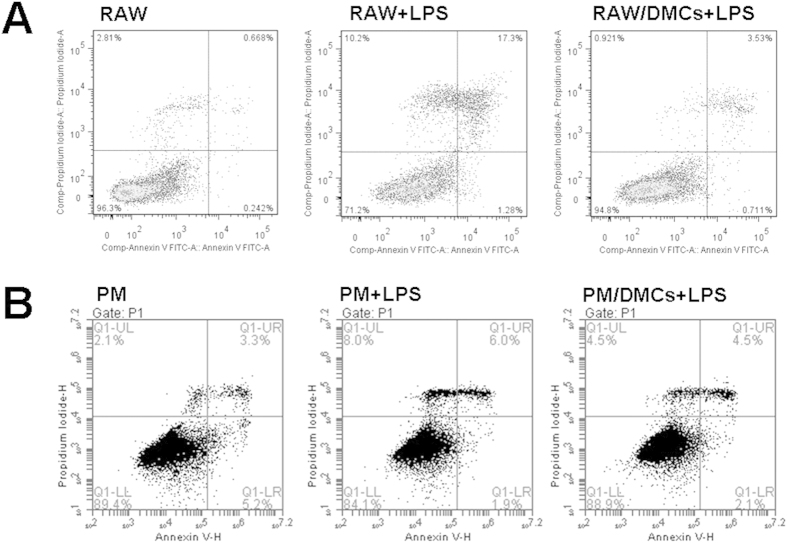
Co-culture with DMCs inhibited apoptosis in macrophages. (**A**) Raw264.7 were stained with Annexin-V-/Propidiumiodid and analyzed for apoptosis in the presence or absence of LPS stimulation. Significantly less apoptosis was observed in co-cultured Raw264.7 cells (Annexin-V-positive, PI-negative cells). RAW: Raw264.7 alone without LPS stimulation; RAW + LPS: Raw264.7 alone with LPS stimulation; RAW/DMCs + LPS: non-contact co-culture of DMCs with Raw264.7 with LPS stimulation. (**B**) Peritoneal macrophages apoptosis. PM: peritoneal macrophages alone without LPS stimulation; PM + LPS: peritoneal macrophages alone with LPS stimulation; PM/DMCs + LPS: non-contact co-culture of DMCs with peritoneal macrophages with LPS stimulation. Results represent 3 independent experiments.

To demonstrate the effect of DMCs on Raw264.7 apoptosis induced by LPS, we repeated our co-culture experiments with PI-/Annexin-V-staining. After 24 h of co-culture and monoculture, we used APC-conjugated Annexin-V and PI to identify the fraction of apoptotic cells (Annexin V positive, PI negative) within the population of co-cultured Raw264.7 in comparison to identically stained Raw264.7 in monoculture. Indeed, co-culture with DMCs decreased the number of apoptotic Raw264.7 by less than one quarter when compared to monocultured Raw264.7 (3.53% vs. 17.3%, Fig. 6A). DMCs also showed protection function on peritoneal macrophages to some extend (Fig. 6B).

### Co-culture with DMCs increased migration activity of macrophages in the presence or absence of LPS stimulation

**Figure 7:**
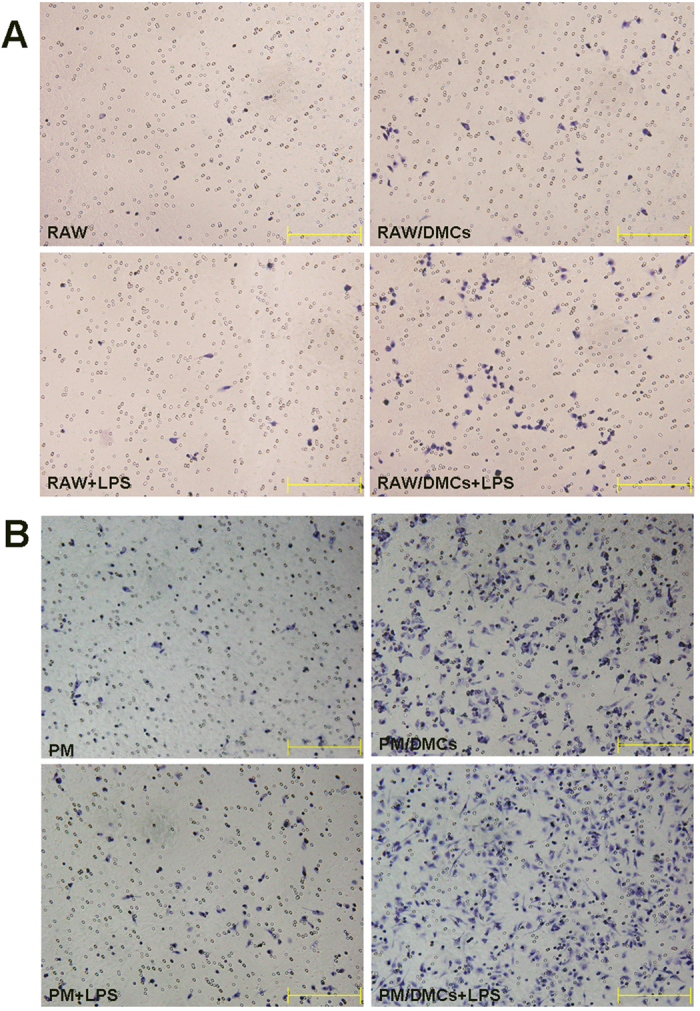
A co-culture experiment showed that DMCs can increase the migration activity of peritoneal macrophages and Raw264.7 macrophages in the presence or absence of LPS. DMCs were co-cultured with macrophages in transwell (8 μm) in non-contact condition. The migration activity of macrophages was measured at 6 h after LPS (1 μg/ml) stimulation. Cells migrating through the membrane were stained by hematoxylin after fixed with 4% paraformaldehyde. (**A**) Raw264.7 macrophages migration. RAW: Raw264.7 alone without LPS stimulation; RAW + LPS: Raw264.7 alone with LPS stimulation; RAW/DMCs: non-contact co-culture of DMCs with Raw264.7 without LPS stimulation; RAW/DMCs + LPS: non-contact co-culture of DMCs with Raw264.7 with LPS stimulation. (**B**) peritoneal macrophages migration. PM: peritoneal macrophages alone without LPS stimulation; PM + LPS: peritoneal macrophages alone with LPS stimulation; PM/DMCs: non-contact co-culture of DMCs with peritoneal macrophages without LPS stimulation; PM/DMCs + LPS: non-contact co-culture of DMCs with peritoneal macrophages with LPS stimulation. Results represent 3 independent experiments. Bar represent 200 μm.

We investigated the migratory response of peritoneal macrophages or Raw264.7 cells to the co-culture with DMCs in the presence or absence of LPS stimulation. As shown in Fig. 7A, DMCs significantly increased the migration activity of Raw264.7 cells both in the presence and absence of LPS stimulation. In detail, the number of migrated Raw264.7 macrophages in culture alone was 0.8 ± 0.8 cells per field and the number of those in co-culture with DMCs was 31.8 ± 14.6 cells per field (p < 0.01) without LPS stimulation. In the presence of LPS stimulation, there was similar trend in the migration activity of monocultured Raw264.7 compared to that of cells co-cultured with DMCs (9.4 ± 8.5 cells vs. 56.8 ± 35.0 cells, p < 0.05). We observed similar change of the migration activity of peritoneal macrophages in co-culture group comparing to peritoneal macrophages alone (Fig. 7B).

### Co-culture with DMCs enhanced phagocytosis of macrophages

**Figure 8:**
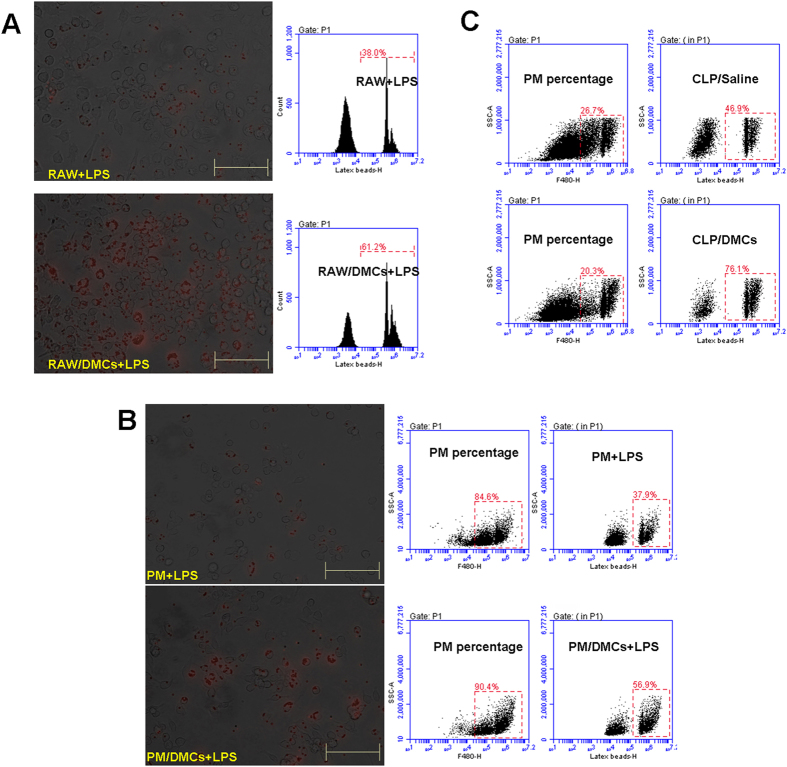
DMCs enhanced phagocytosis of macrophages. (**A**) macrophages Raw264.7 were co-cultured with DMCs for 4 h, then stimulated by LPS (1 μg/ml). And then Raw264.7 cells were incubated with fluorescent latex beads for 12 h. Phagocytosis ability were analyzed by fluorescence microscope and FACS. RAW + LPS: Raw264.7 alone with LPS stimulation; RAW/DMCs + LPS: non-contact co-culture of DMCs with Raw264.7 with LPS stimulation. (**B**) Peritoneal macrophages were treated as Raw264.7, then incubated with fluorescent latex beads for 4 h. Phagocytosis ability were analyzed by fluorescence microscope. Peritoneal macrophages were identified using a PE anti-mouse F4/80 antibody and were measured by FACS. PM + LPS: peritoneal macrophages alone with LPS stimulation; PM/DMCs + LPS: non-contact co-culture of DMCs with peritoneal macrophages with LPS stimulation. (**C**) Phagocytosis analysis *in vivo*: The CLP mice were made and treated with saline or DMCs as described above. One day after CLP, fluorescent latex beads were injected into the peritoneal cavity of CLP mice. Mice were sacrificed 2 hours after injection. Peritoneal cells were stained by PE anti-mouse F4/80 and analyzed by FACS. Results represent 3 independent experiments. Bar represent 100 μm.

To explore the effect of DMCs on the phagocytosis of macrophages, fluorescent latex beads were utilized to test phagocytosis of macrophages. Results shown in Fig. 8A,B clearly indicated that DMCs coculture leads to an increase of phagocytic activity in peritoneal macrophages and RAW 264.7 cells, as evidenced by the red fluorescent dots observed inside the macrophages by microscopic analysis. This effect is further demonstrated in assay by flow cytometry (Fig. 8A,B).

A typical experiment shows that the fluorescent beads could be detected in about 56.9% (peritoneal macrophages) and 61.2% (RAW 264.7 cells) of cells cocultured with **DMCs**. In contrast, only about 37.9% (peritoneal macrophages) and 38% (RAW 264.7 cells) of monocultured cells exhibit fluorescence. *In vivo* phagocytosis assessment indicated that the fluorescent beads could be detected in about 76.1% of peritoneal macrophages in DMCs treated group. In contrast, only about 46.9% of peritoneal macrophages in saline control group exhibit fluorescence (Fig. 8C). Taken together, these results indicated that Co-culture with DMCs enhanced phagocytosis of macrophages *in vitro* and *in vivo*.

### MiRNA microarray analysis

**Figure 9:**
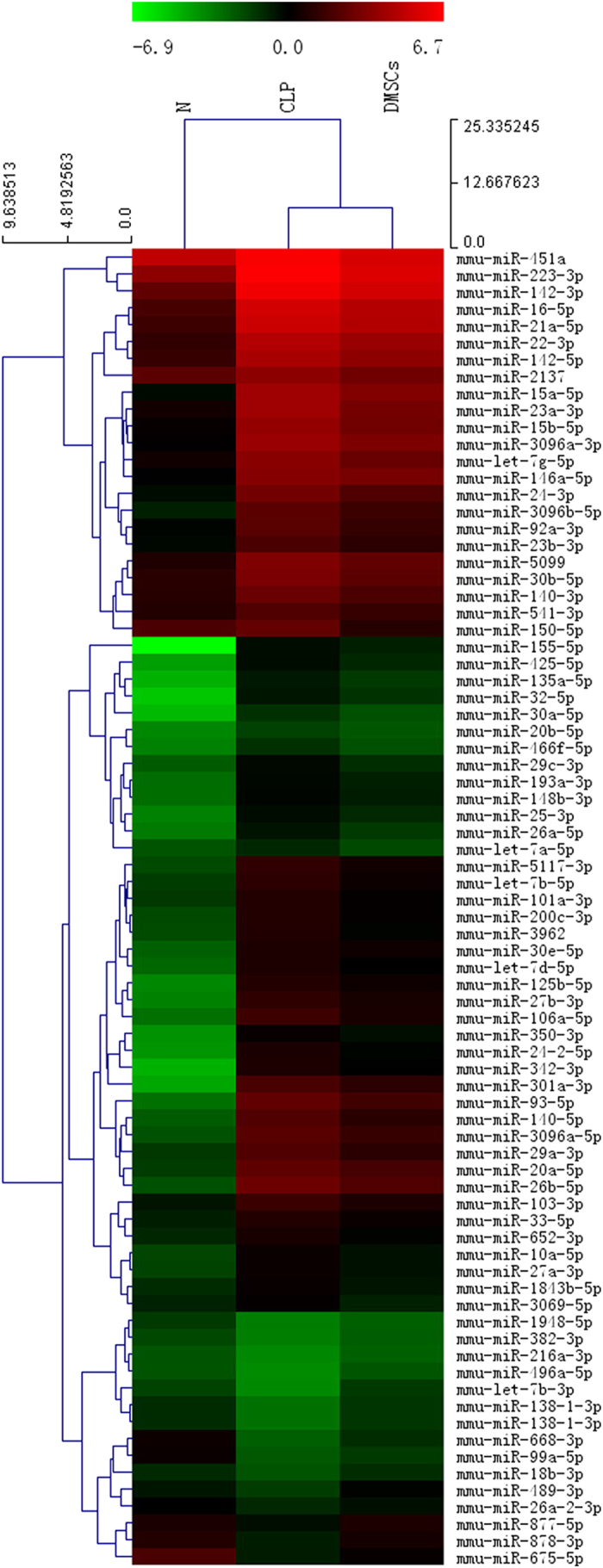
Expression profiles of miRNA. We performed microarray analysis for miRNA expression with RNA extracts from murine peritoneal macrophages. We visualized the relative fold change expression of experimental conditions compared with Sham/saline using a heat map: green = underexpression; black = no change; red = overexpression.

To identify the molecular changes associated with decreased inflammation in CLP-injured mice treated with DMCs, we analyzed the miRNA expression profiles from peritoneal macrophages collected at 24 hours: sham/saline, CLP/saline, and CLP/DMCs. Of mouse miRNAs examined using microarray hybridization, 171 miRNAs were 2.0 fold down regulated and 175 miRNAs were 2.0 fold up regulated in CLP/saline mice compared with sham/saline mice. Of these differentially expressed miRNAs,15 miRs were upregulated and 62 were downregulated after MSC treatment(cut-off with a threshold of ≥ 1.2 fold or ≤−1.2 fold, Fig. 9). Among these 77 dysregulated miRs, we identified 12 altered miRNAs that were 1.5 fold regulated by DMCs and average intensity >100, including miR-23a-3p, miR-3069-5p, miR-26a-5p, miR-142-3p, miR-21a-5p, miR-223-3p, miR-16-5p, miR-22-3p, let-7g-5p, let-7b-5p, miR-878-3p, miR-489-3p.

### Bioinformatics interpretation revealed the GOs and KEGG signaling pathways regulated by miRNAs

**Figure 10:**
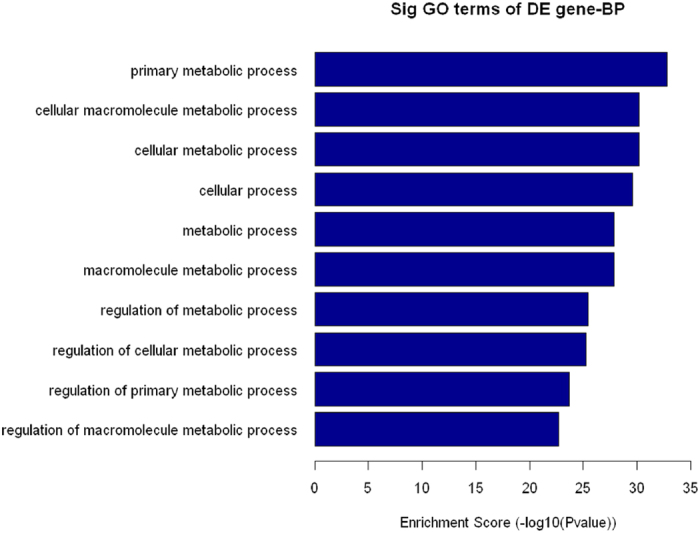
Enriched GO terms in the biological process category among differentially expressed miRNAs associated with DMCs treatment during sepsis. After miRNA microarray assay, significantly enriched GO analysis in the biological process category was performed on differentially expressed genes in the CLP-induced sepsis mice using DAVID (P < 0.01). Only the top ten GO terms are listed here.

Target genes regulated by these differentially expressed miRNAs were predicted using TargetScan Mouse, Mirbase, and miRanda. For these 12 differentially expressed miRNAs, TargetScan predicted 5,652, Mirbase predicted 9,059 and miRanda predicted 29,026 target genes. Of these, 748 target genes were predicted under all three systems. Gene ontology (GO) analysis in the Database for Annotation, Visualization and Integrated Discovery (DAVID) was performed for these miRNAs using the predicted gene targets^21^. Functional analysis revealed that the ten most common GO categories were primary metabolic processes, cellular macromolecule metabolic processes, cellular metabolic processes, cellular processes, metabolic processes, macromolecule metabolic processes, regulation of metabolic processes, regulation of cellular metabolic processes, regulation of primary metabolic processes and regulation of macromolecule metabolic process (Fig. 10). These analyses suggest that cellular miRNAs may regulate cellular metabolic processes associated with DMCs treatment during sepsis, either directly or indirectly.

Further analysis demonstrated that miRNAs dysregulated in CLP animals and DMCs treatment were predicted to target mRNAs highly enriched in 42 KEGG pathways (P < 0.05). The top ten were shown in Table 1. Among the list of high-enrichment KEGG pathways of these miRNA targeted genes, the immune-related signaling pathways were significantly prominent, such as the Fc gamma R mediated phagocytosis, transforming growth factor-β signaling pathway, MAPK signaling pathway, cytokine-cytokine receptor interactions, and Jak-STAT signaling pathway. These data indicate that DMCs transplantation modulates inflammatory reaction during sepsis, at least in part, by altering miRNA expression involved in the immune-related signaling pathways.

## Discussion

Skin is the largest organ in the body and is easily accessed with minimal harm to the donor. In this regard, dermis is considered to be one of the best autologous source organs from which to harvest stem/progenitor cells for therapeutic applications, not only in the replacement of skin, but also for other organs^22^. Particular for the patients with traumatic injuries, such as burns, wounds and sepsis, they require an off the shelf product that is immediately available which lends itself better to allogeneic cell products. A number of investigations including our previous work have revealed the existence of resident self-renewing, MSC-like multipotency cells in the dermal skin of rodents, rats and humans^23^,^24^. We have confirmed the multilineage differentiation of DMCs and about 72.6% cells express surface marker CD105. Further, recent studies have demonstrated that *in vitro* culture may alter the functional activities of cells and it is believed that cells isolated from the natural microenvironment can better mimic their properties *in vivo* than the commonly used 2D cultures^14^. Recently, there is emerging studies to use noncultured mesenchymal cells for the regenerative studies. In this study, we firstly used non-cultured DMCs to treat a clinically relevant mouse model of CLP-induced polymicrobial sepsis and demonstrated that non-cultured DMCs could reduce mortality in sepsis mice. We find that DMCs injected intravenously migrate to and accumulate in the inflamed ileocecal region; DMCs administration diminishes the pathological severity of CLP-induced sepsis; DMCs modulate the circulating level of inflammatory cytokines; and coculture with DMCs leads to modulation of inflammatory cytokine secretion, increase in migration and phagocytosis, and inhibition of the apoptosis of macrophages induced by LPS.

Many studies have shown that intravenous and intraperitoneal delivery of MSCs is efficacious because of their capacity for homing to injured tissues, such as the lung^25^, myocardium^26^, brain^27^, liver^28^, and kidney^29^. In our study, we demonstrated that intravenously delivered DMCs migrated towards the inflamed ileocecal region and stayed there for more than 8 days. However, in the non-inflamed tissues, such as the proximal colon, no labeled DMCs were observed. We also did not find labeled DMCs in the lung or kidney, and rarely found labeled DMCs in the liver 4d after operation. This feature of homing to and colonizing in injury sites makes DMCs particularly appealing in the treatment of the CLP-induced sepsis. We revealed that cocultured with DMCs significantly increased migration activity of macrophages. One essential step in host defense is macrophages migrating from the blood to the sites of inflammation, which is important in recognizing and eliminating the harmful microorganisms of the host^30^. The increased migration activity of macrophages may partly explain the effect of DMCs on CLP-induced sepsis mice.

An important finding in our model is that injection of DMCs into septic mice reduced the expression of proinflammatory cytokines IL-1β and IL-6, while modulated serum IL-4, IL-5, and IFN-γ levels near to wild-type levels relative to saline-treated control mice. DMCs also show the ability to modulate the sepsis associated cytokine secretion, which is coincident with the properties of MSCs isolated from different tissues. However, serum concentrations of IL-10, which was found playing an important role in bone marrow MSC-mediated protection against sepsis^31^, was not increased by DMCs treatment in our experiments. These differences may be due, in part, to the intrinsic immunoregulation properties of various cell sources. The findings by Fletcher and Mei SH are also similar to ours regarding IL-10 expression after cell therapy of sepsis^10^,^32^. Meanwhile, we observed an increasing of serum IL-5 level in DMCs treated septic mice. Linch and his colleagues’ results demonstrated that IL-5 was protective during sepsis through improving survival and host control of infection^33^. Previous researches have not reported the serum IL-5 concentrations altered by other MSCs treatment. We infer that the immunoregulation properties of DMCs differ from that of other MSCs. Cell-cell contact appears to be a key mechanism by which MSCs modulate immune effector cells such as macrophages and T cells^34^. Our results showed cell-contact lead to a more robust effect on levels of IL-6 and IL-10, which was coincident with Nemeth and his colleagues’ results^31^. They showed that macrophages produced significantly more IL-10 in response to LPS stimulation than when they were cultured in transwell plates without direct contact with the BMSCs or exposed to BMSC-conditioned medium. The underlying mechanisms of cell-cell contact modulatory effect on inflammatory cytokines release are far away from elucidated and need more further investigation.

In our study, DMCs treatment reduced apoptotic cells in the lung to the levels observed in sham-operated animals. And coculture with DMCs cuts the number of apoptotic Raw264.7 by less than one quarter when compared to monoculture of Raw264.7 *in vitro*. These results indicate that DMCs have similar anti-apoptotic functions comparing with MSCs, which have been demonstrated by other reasearches^32^. Meanwhile, we were surprised to find that DMCs treatment also improved macrophage phagocytosis *in vivo* and *in vitro*, suggesting that DMCs could play a role in modulating bacterial clearance. Although the exact mechanism of increased phagocytosis warrents further investigation, the miRNAs expression microarray analysis performed in our study revealed up-regulation of pathways associated with Fc gamma R mediated phagocytosis. On the other hand, as recent publication shows that IL-5 plays a role in modulating phagocytosis^33^, increased macrophage phagocytosis could partly be explained by increased IL-5 level after DMCs treatment.

Recent publications have provided convincing evidence that a number of miRNAs are involved in the regulation of immunity. More importantly, researchers have reported that cellular miRNAs play key regulatory roles during sepsis and that altered cellular miRNA expression in response to infection may be an important determinant of sepsis^20^,^35^,^36^. Because peritoneal macrophages play an important role during sepsis, we used microarray analysis to characterize the relative expression of miRNAs in peritoneal macrophages during sepsis. We found that the inflammatory miRNAs expression profile from the DMCs treated CLP mice was more similar to the profile of the sham/saline mice than that of CLP mice, providing further evidence that DMCs treatment “normalized” inflammatory miRNAs activity in sepsis. We next focused our studies on miRNAs dysregulated during sepsis that returned near to basal levels after DMCs treatment. biology bioinformatic algorithms such as TargetScan and miRanda are reliable tools for predicting mRNA targets of miRNAs. Then, we compiled and applied this target gene pool to GO and KEGG pathway analysis using the DAVID Functional Annotation tool to provide statistical reference for inferred associations between target mRNAs and biological pathways. The ten most common GO categories were primary metabolic processes, cellular macromolecule metabolic processes, cellular metabolic processes, cellular processes, metabolic processes, macromolecule metabolic processes, regulation of metabolic processes, regulation of cellular metabolic processes, regulation of primary metabolic processes and regulation of macromolecule metabolic process. These analyses suggest that cellular miRNAs may regulate cellular metabolic processes during DMCs treatment, either directly or indirectly. We next applied the miRNA-targeted gene pool to KEGG pathway analysis, which identifies frequent and enriched pathways. Functional analysis showed the differentially expressed miRNAs to be involved in many immune-related signaling pathways, such as the Fc gamma R mediated phagocytosis, MAPK signaling pathway, cytokine-cytokine receptor interactions, and Jak-STAT signaling pathway associated with DMCs treatment during sepsis. we speculated that post-transcriptional modifications such as microRNA regulation of the immunomodulatory effects after DMCs treatment could possibly fit in a mechanistic framework that will explain the molecular basis of DMCs therapy in sepsis. The present data provide candidates for further analysis of miRNAs in DMCs-based therapy and for the development of potential biomarkers and therapeutic targets for sepsis.

## Conclusion

In summary, this study demonstrates that non-cultured DMCs administration provides a beneficial effect to improve survival in CLP-induced sepsis. This effect is partly mediated by the ability of DMCs to home to sites of injury, to reduce the inflammatory response, to inhibit apoptosis, to stimulate macrophage migration and phagocytosis. Our further findings suggest that DMCs treatment modulates the beneficial cytoprotective effects exhibited during sepsis, at least in part, by altering miRNA expression. The modulated miRNAs might regulate biological processes of cells during sepsis. The predicted target genes of these differentially expressed miRNAs are involved in immune responses in the host. These discoveries provide important evidence that non-cultured DMCs therapy has a specific anti-inflammatory effect on the mouse model of sepsis, and provide the basis for the development of a new therapeutic strategy for managing clinical sepsis, which remains a threat to critically ill patients and carries a high morbidity and mortality.

## Additional Information

**How to cite this article**: Wang, Y. *et al.* Non-cultured dermal-derived mesenchymal cells attenuate sepsis induced by cecal ligation and puncture in mice. *Sci. Rep.* **5**, 16973; doi: 10.1038/srep16973 (2015).

## Figures and Tables

**Table 1:** Kyoto Encyclopedia of Genes and Genomes pathway analysis based on miRNA-targeted genes (top ten).

Definition	Fisher-Pvalue	Enrichment_Score	Genes
Fc gamma R-mediated phagocytosis	7.12165E-06	5.147419	AMPH//ARPC5//CFL2//LIMK2//MAP2K1//MARCKS//MARCKSL1//PPAP2A//PRKCD//RAF1//VAV2//WASF1//WASL
Proteoglycans in cancer	7.29608E-05	4.136911	ANK3//CASP3//CAV3//CTSL//DDX5//ELK1//FAS//FASL//FGFR1//HGF//HOXD10//MAP2K1//MRAS//PDCD4//PPP1CB//RAF1//RDX//RRAS//TIAM1//VAV2
TGF-beta signaling pathway	8.15546E-05	4.088551	ACVR1C//ACVR2A//CHRD//E2F5//FST//PITX2//PPP2CB//PPP2R1A//SMAD1//SMAD7//TGFBR1
MAPK signaling pathway	0.000106936	3.970875	CASP3//DUSP2//ELK1//FAS//FASL//FGFR1//MAP2K1//MAP2K3//MAP3K1//MAP3K11//MAP4K3//MAP4K4//MAPK10//MEF2C//MKNK1//MRAS//NTF3//RAF1//RPS6KA6//RRAS//TGFBR1
Regulation of actin cytoskeleton	0.000113565	3.944756	APC//ARPC5//CFL2//FGD1//FGFR1//GIT1//LIMK2//MAP2K1//MRAS//MYH10//PPP1CB//RAF1//RDX//RRAS//TIAM1//VAV2//VCL//WASF1//WASL
Hippo signaling pathway	0.000624167	3.204699	APC//CCND3//LLGL2//NF2//PPP1CB//PPP2CB//PPP2R1A//SMAD1//SMAD7//SOX2//TGFBR1//YWHAG//YWHAH//YWHAQ
African trypanosomiasis	0.000953456	3.0207	FAS//FASL//IL10//IL12A//IL6//VCAM1
Hepatitis B	0.001177536	2.929026	CASP3//CCNE1//ELK1//FAS//FASL//IL6//MAP2K1//MAP3K1//MAPK10//PTK2B//RAF1//TGFBR1//YWHAQ
Pathways in cancer	0.001213597	2.915925	APC//CASP3//CCNE1//CKS2//CSF1R//CUL2//EGLN2//FAS//FASL//FGFR1//HGF//HSP90B1//IL6//MAP2K1//MAPK10//RAF1//RARB//RET//RXRG//TFG//TGFBR1//TPM3
Cytokine-cytokine receptor interaction	0.001655934	2.780957	ACVR2A//CCL1//CCL7//CCR7//CNTFR//CSF1R//CXCL5//FAS//FASL//HGF//IFNGR2//IFNK//IL10//IL12A//IL13//IL6//PRLR//TGFBR1//TNFRSF13C
